# The Human Heart

**Published:** 1889-02

**Authors:** 


### THE HUMAN HEART.

A curious calculation has been made by Dr. Richardson, giving the work of the heart in mileage. Presuming that the blood was thrown out of the heart at each pulsation in the proportion of 69 strokes per minute, and at the assumed force of 9 feet, the mileage of the blood through the body might be taken at 207 yards per minute, 7 miles per hour, 168 miles per day, 61,320 miles per year, or 5,150,880 miles in a lifetime of eighty-four years. The number of beats of the heart in the same long life would reach the grand total of 2,869,776,000.—*Medical World.*

